# Preparation of Fibrillated Cellulose Nanofiber from Lyocell Fiber and Its Application in Air Filtration

**DOI:** 10.3390/ma11081313

**Published:** 2018-07-29

**Authors:** Jin Long, Min Tang, Yun Liang, Jian Hu

**Affiliations:** 1 State Key Laboratory of Pulp and Paper Engineering, South China University of Technology, Guangzhou 510641, China; lj_king@139.com (J.L.); liangyun@scut.edu.cn (Y.L.); ppjhu@scut.edu.cn (J.H.); 2 Department of Mechanical Engineering, University of Minnesota—Twin Cities, Minneapolis, MN 55455, USA

**Keywords:** cellulose nanofiber, Lyocell fiber, PM_2.5_, filter paper

## Abstract

Ambient particulate matter less than 2.5 μm (PM_2.5_) can substantially degrade the performance of cars by clogging the air intake filters. The application of nanofibers in air filter paper can achieve dramatic improvement of filtration efficiency with low resistance to air flow. Cellulose nanofibers have gained increasing attention because of their biodegradability and renewability. In this work, the cellulose nanofiber was prepared by Lyocell fiber nanofibrillation via a PFI-type refiner, and the influence of applying a cellulose nanofiber on filter paper was investigated. It was found that the cellulose nanofibers obtained under 1.00 N/mm and 40,000 revolutions were mainly macrofibrils of Lyocell fiber with average fiber diameter of 0.8 µm. For the filter papers with a different nanofiber fraction, both the pressure drop and fractional efficiency increased with the higher fraction of nanofibers. The results of the figure of merit demonstrated that for particles larger than 0.05 µm, the figure of merit increased substantially with a 5% nanofiber, but decreased when the nanofiber fraction reached 10% and higher. It was concluded that the optimal fraction of the cellulose nanofiber against PM_2.5_ was 5%. The results of the figure of merit were related to the inhomogeneous distribution of nanofibers in the fibrous structure. The discrepancy of the theoretical and measured pressure drop showed that a higher nanofiber fraction led to a higher degree of fiber inhomogeneity.

## 1. Introduction

Clean air is considered a basic requirement for modern society. However, the air quality deterioration and the increasing air pollution has aroused widespread public concerns. The U.S. Environmental Protection Agency developed the first standard for particulate matter less than 2.5 μm (PM_2.5_) in 1997, as a result of concern over the health effects of fine particles in the air [1]. Some major cities in China and India, for example, Beijing and New Delhi, are frequently exposed to severe PM_2.5_ pollution. Besides the health effects, ambient particles can affect human life in other ways. One important effect is that it can substantially degrade the performance of cars, as the combustion engine of a car needs to ingest large quantities of air. The main impact of PM_2.5_ on filtration performance, particularly in combination with moisture [2,3], is that it will clog the filters in a shorter time than conventional dust [4]. Fine particles are mainly collected by interception and diffusion [5], and they form dendritic particle structures that contribute to airflow resistance substantially [6]. As the initial efficiency of the traditional filter paper against fine particles is low, the PM_2.5_ pollution challenges the filter performance and consequently increases the potential damage for the vehicle engines. As a result of the increasing problems of fine particle pollution, the requirement of better filter performance is more demanding. In addition, the global commitment to cleaner energy and less energy usage is motivating the filtration industry to develop a filter media with less resistance to air flow, thereby reducing energy usage.

Most of the filter media for engine intake filtration are in the form of wet-laid paper [7]. Filter paper is pleated so that the filtration area and dirt-holding capacity could be increased many times in a small space left for air filter [8]. Cellulose-type filter paper, made of fibers with diameters larger than 10 microns, are often used in engine air filtration. To improve filtration performance, the application of nanofiber has gained lots of attention. Many theoretical results and experimental data have shown that the filtration efficiency could be dramatically improved with a low resistance to air flow, due to the slip effect at the gas-fiber interface and the large specific surface area of the nanofiber [9,10,11,12]. There are two popular methods of applying nanofibers. One method is to laminate membrane-like nanofiber layer on top of the micrometer fiber by electrospinning, which will result in rapid clogging by the deposited particles, as described by Leung et al. [13]. Another method of producing the nanofiber composite is to add nanofibers (mostly glass wool fiber) in the wet-laid forming process. The nanofibers are well dispersed among the micrometer fibers, which can solve the problems of laminated nanofiber filter paper, such as weak strength and short service life. Although glass wool fiber shows an excellent filtration performance, it causes problems for the used filter paper when being disposed, as glass fiber is non-biodegradable and cannot be burned. Moreover, there is a health concern over glass wool fiber. Glass wool fibers, such as E-glass and 475 glass fibers, are considered by International Agency for Research on Cancer, IARC, as possible carcinogens to humans (Group 2B). There is a demand to find an alternative nanofiber that is human friendly and suitable for a wet-laid process.

As the search for efficient bio-based materials is the main challenge of the next decades [14], the development of cellulose nanofibers has gained increasing attention [15,16,17,18,19,20,21,22,23], because of their high strength, biodegradability, and renewability [24]. The extensive separation of cellulose fibers into nanofibers can be achieved if conventional refining methods are applied well beyond the levels used in paper making [25,26,27]. Among either natural or man-made cellulose fibers, the Lyocell fibers differ from others in its high crystallinity, high longitudinal orientation of crystallites, and low lateral cohesion between fibrils [28,29,30]. Lyocell is an environmentally benign man-made fiber, because it is manufactured by cellulose dissolution in nontoxic N-methyl morpholine N-oxide instead of toxic carbon disulfide, which can be almost totally recycled [31]. In the swollen state, the Lyocell fiber has an extensive tendency to fibrillate [32]. Several studies have investigated the fibrillation of the Lyocell fibers by different fibrillating equipment (e.g. metal balls with tumbling [33] and crockmeter [34]), but the properties of the fibrillated fibers that were obtained from these studies were highly dependent on their fibrillating equipment. The study of Lyocell fibrillation on a more reproducible machine (e.g. PFI-type pulp refiner [35]) was necessary. Although the fibrillated Lyocell nanofiber is a very interesting material for eco-friendly filter paper, not much literature has been reported about its application in engine filtration. Unlike other applications, the nanofiber in filter paper has its unique way of designing a fibrous structure. Tang et al. [36] and Choi et al. [37] found that adding more nanofibers did not lead to a better filtration performance, as expected by the conventional filtration theory. The study of the nanofiber structure is critical to optimize the performance of filter paper. More studies of using nanofibers in filtration are needed. The goal of this work is to prepare Lyocell cellulose nanofiber suitable for wet-laid filter paper, and to explain the microstructure changes in the fibrillation process using the PFI-type refiner. The optimization of the nanofiber mixing fraction will also be studied based on the results of the filtration test against the PM_2.5_ and analysis of the fibrous structure.

## 2. Materials and Methods 

### 2.1. Nanofibrillation of Lyocell Fiber

Lyocell fiber (TENCEL^TM^, Lenzing AG, Lenzing, Austria) with a linear mass density of 1.7 dtex and length of 4 mm, as shown in Table S1, was used to prepare the cellulose nanofiber. As the Lyocell fibers were continuously drawn from the spinneret pierced with small holes in the manufacturing process, the Lyocell fibers possessed a uniform diameter (12 µm in this study) and circular cross section.

The nanofibrillation of the Lyocell fiber was conducted on a PFI-type pulp refiner (model: mark VI, Hamjern Maskjn, Hamar, Norway). The PFI refiner is a machine with a high reproducibility for use in the laboratory under standardized conditions [35]. The key elements of the PFI refiner were a stainless steel roll with chiseled bars and a cylindrical container with a smooth interior wall. The roll and the container were independently driven in the same direction, with a constant difference of peripheral speed, which resulted in mechanical effects such as shear and compression for fiber fibrillation. In this study, the refining followed the procedures in the TAPPI T248 [38] standard. The Lyocell fibers were weighed and then mixed with distilled water to form a slurry with a fiber consistency (concentration) of 10%. The fiber slurry was pressed evenly to the interior wall of the container. In the refining process, the roll and the cylindrical container rotated in the same direction at 1440 rpm (revolutions per minute) and 720 rpm, respectively. To study the impact of the refining pressure on the fibrillation, the roll was pressed against the container wall under two pressures, 1.00 N/mm and 3.33 N/mm (recommended pressure in TAPPI T248). For each refining pressure, the refining procedure was stopped and the fibrillated fiber was obtained when the revolution number of refiners reached to 5000, 10,000, 20,000, and 40,000, respectively. 

### 2.2. Characterization of Lyocell Nanofiber

The morphology of the fibrillated Lyocell fiber was observed by an optical microscope (model: BX51TF, Olympus Corporation, Tokyo, Japan). The images were captured by a photomicrographic system equipped with the microscope, which can give a broad view to analyze the fibrillation and length of the Lyocell fiber. Based on the analysis of the microscopy images, a preferred refining pressure was selected for the subsequent studies. After determining the preferred refining pressure, the main influencing factor of the fibrillation process was the number of revolutions. The fibrillation and nanofiber morphology under different revolution numbers were observed by a scanning electron microscope (model: EV018, Carl Zeiss AG, Jena, Germany). 

As the fibrillated Lyocell fiber consisted of microfiber and nanofiber, it was hard to obtain an average fiber diameter for the fibrillated Lyocell fiber. The beating degree and specific surface area were used to characterize the fiber diameter after fibrillation. The beating degree was measured by Schopper-Riegler beating tester (model: ZJG-100, Changchun Yueming Small Tester Co., Ltd, Jilin, China) following ISO 5267-1 [39]. The specific surface area was tested using the Brunauer–Emmett–Teller surface area analyzer (model: SA 3100, Beckman Coulter, Brea, CA, USA). Based on the specific surface area, the average diameter of the nanofiber, *d_f_* (µm), can be estimated by Equation (1), as follows:

(1) $$d_{f}=\frac{4000}{\rho S}$$

where, *ρ* is the fiber density, kg/m^3^, and *S* is the specific surface area, m^2^/g.

The weighted average fiber length (length-weighted) and fine fiber content were measured using the fiber quality analyzer (model: FS-300, Metso Automation, Vantaa, Finland). After the characterization and evaluation of the fibrillated Lyocell fiber, an optimal refining condition was selected and the nanofiber obtained from this condition was used to prepare the nanofiber composite filter paper.

### 2.3. Preparation of Nanofiber Composite Filter Paper

The fibers used in commercial filter paper for engine air intake were wood fiber and synthetic fiber. In this study, softwood fiber (from Suzano Pulp and Paper Inc., São Paulo, Brazil) and PET (polyethylene terephthalate) fiber (dimension: 1.7 dtex × 5 mm, Kuraray Co. Ltd, Tokyo, Japan) were selected to mix with the Lyocell nanofiber. The weight fraction of each fiber in the filter paper can be seen in Table 1. The basic weight for all of the samples was 105 ± 2 g/m^2^. 

The preparation of the nanofiber composite filter paper was done according to the procedures provided by ISO 5269-1:2005 [40]. The fibers were weighed by an electronic balance with 1.0 mg accuracy, and were mixed together with 2.0 L water. The mixture was dispersed in the standard fiber disintegrator (model: P95568, PTI GmbH, Laakirchen, Austria) under 3000 rpm for 200 seconds. The well-dispersed fiber suspension was transferred to the handsheet former (model: RK3AKWT, PTI GmbH, Laakirchen, Austria). Then, 5.0 L of water was added into the handsheet former to decrease the fiber consistency and to improve the handsheet formation. After the agitation is completed, the fiber suspension was drained through a metallic screen (120 mesh). The fibers were retained on the circular screen, with a diameter of 200 mm, and thus a wet handsheet was formed. Finally, the wet handsheet was carefully transferred to the dryer (Speed Dryer, PTI GmbH, Laakirchen, Austria) and dried under 105 ℃ for 20 minutes. The dryer was carefully selected to minimize the compression on the porous structure of the handsheet and to keep the handsheet flat during the drying process. After the preparation of the filter media, the thickness was measured according to Tappi T411 [41], and the mean flow pore size was measured by a capillary flow porometer (model: CFP 1100, Porous Material Inc., New York, NY, USA) using a wet up/dry down procedure based on ASTM F316-03 [42]. The porosity (*ε*) of the filter paper can be calculated from the basic weight (*BW*, g/m^2^), thickness (*l*, mm), and fiber density (*ρ_f_*, kg/m^3^), as shown in Equation (2). The preparation and testing for each type of paper were repeated five times.

(2) $$\varepsilon =1-\frac{BW}{\rho_{f}l}$$

### 2.4. Filtration Test of Nanofiber Composite Filter Media

To evaluate the capability of capturing the PM_2.5_, the fractional efficiency against the particles ranging from 30 nm to 2 µm was measured. This range covered most of the ambient PM_2.5_, as indicated by the particle size distributions of Thielke et al. [43]. The fractional efficiency was measured using monodisperse potassium chloride (KCl) particles with diameter of 30 nm, 50 nm, 80 nm, 100 nm, 150 nm, and 200 nm, as described by Tang et al. [44], and polydisperse KCl particles ranging from 400 nm to 2 µm, as described in the following section.

**Figure 1:**
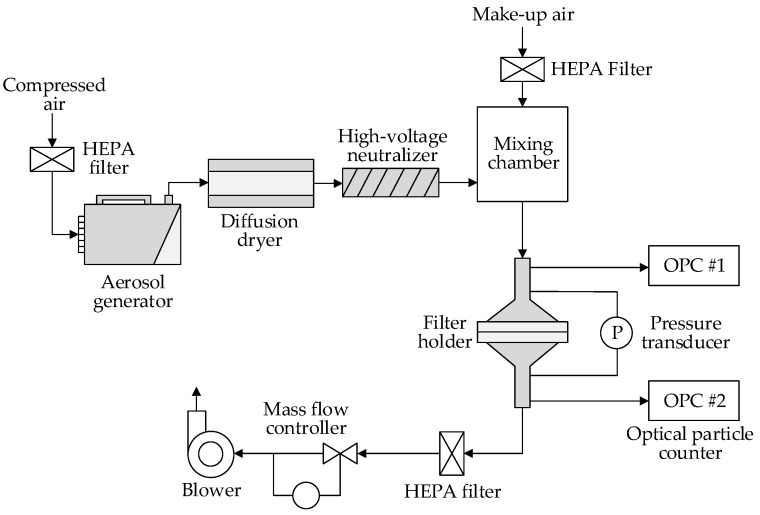
Schematic of filtration test setup.

The polydisperse KCl particles were generated by an aerosol generator (model: RBG 2000, Palas GmbH, Karlsruhe, Germany) using a 12 wt % KCl solution. The KCl solution was broken into fine droplets by compressed air in the aerosol generator. The droplets were dried using a silica gel desiccator in the diffusion dryer, and they became solid particles. Then, the particles were neutralized using ion stream from a high-voltage neutralizer (model: 3088, TSI Inc., Shoreview, MN, USA), in order to achieve the Boltzman charge equilibrium. After neutralization, the KCl particles were ready for the filtration test. All of the compressed air used in this system was filtered by a high efficiency particulate air (HEPA) filter, in order to obtain clean air (see Figure 1).

The filter paper was mounted in the filter holder with the test area of 100 cm^2^. The upstream and downstream of the filter holder were connected to a pressure transducer (Model: 166, Alpha Instruments Inc., Acton, MA, USA) and two optical particle counters (OPC, model: 3330, TSI Inc., Shoreview, MN, USA). The pressure transducer measured the pressure drop across the filter paper under a certain testing velocity. The optical particle counters sampled the air constantly from the test flow and measured the particle number concentration of the upstream and downstream air. The measurement was based on ISO/TS 19713-1:2010 [45]. The upstream number concentration, *N_up,i_* (#/cm^3^), and downstream concentration, *N_down,i_* (#/cm^3^), of the particles with a diameter of *d_i_* were used to calculate the filtration efficiency, *E_i_*, as shown in Equation (3). To obtain reliable results, the particle concentration should be appropriate to meet the minimum upstream counts (≥500) and should avoid the effect of the particle deposition on the filtration performance. The particle mass concentration in this study is 10 mg/m^3^, under which the pressure drop would not change for 30 minutes.

(3) $$E_{i}=1-\frac{N_{down,i}}{N_{up,i}}$$

A good filter paper is one that gives a high collection efficiency with a low pressure drop. A useful criterion for comparing filter papers is the figure of merit [44], *FOM_i_* (Pa^‒1^), of particles with a diameter of *d_i_*, as shown in Equation (4).

(4) $$FOM_{i}=\frac{\ln(1-E_{i})}{\Delta p}$$

where Δ*p* is the pressure drop, Pa.

A mass flowmeter (Model: 4043, TSI Inc., Shoreview, MN, USA) and a ball valve on the downstream were used to control the test flow rate. The face velocity across the filter paper was 10.0 cm/s, which was frequently used in the air filter test for the engine intake system. The temperature of the ambient air was 21 °C with a relative humidity less than 10%.

## 3. Results and Discussion

### 3.1. Effect of Refining Pressure on Fibrillation of Lyocell Fiber

**Figure 2:**
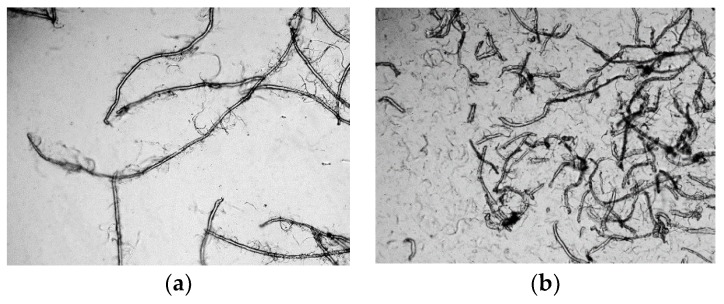
Microscopic images (×100) of fibrillated the Lyocell fiber under refining pressure of 3.33 N/mm: (**a**) 10,000 revolutions; (**b**) 40,000 revolutions.

**Figure 3:**
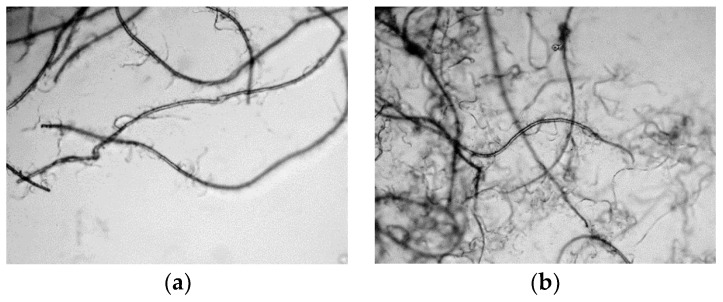
Microscopic images (×100) of the fibrillated Lyocell fiber under refining pressure of 1.00 N/mm: (**a**) 10,000 revolutions; (**b**) 40,000 revolutions.

The microscopic images of the fibrillated Lyocell fiber under different refining pressure and revolutions can be seen in Figure 2 and Figure 3. The magnification ratio (100:1) for each image was the same, which meant that the fiber size in the microscopic images was comparable. As can be seen, when the refining pressure was standard, 3.33 N/mm, the Lyocell fiber could be effectively fibrillated. When the number of revolutions reached 10,000, the fine nanofibers were fibrillated from the bulk Lyocell fiber. However, when the number of revolutions reached 40,000, the Lyocell fibers were cut into short fibers, and were compared with Figure 2a. It demonstrated that under a refining pressure of 3.33 N/mm, the energy from the refiner was largely used to cut the Lyocell fiber instead of the fiber fibrillation. The massive fragmented fibers indicated that the fibrillation under this refining pressure was unsatisfactory.

When the refining pressure was 1.00 N/mm, the fiber fibrillation was more satisfactory. Although the fibrillation process was slower under the refining pressure of 1.00 N/mm, as can be seen in Figure 2a and Figure 3a, the Lyocell fiber was less likely to be cut into short fibers. When the number of revolutions reached 40,000, abundant nanofibers were produced, but the Lyocell fibers were still long. The energy from the refiner was largely used to fibrillate the Lyocell fiber instead of cutting the fiber. As the energy consumption of the PFI refiner was related to the number of revolutions, it was apparent that a refining pressure of 1.00 N/mm was more efficient for the Lyocell fiber fibrillation. Based on the microscopic observation, the preferred refining pressure for the Lyocell fiber was 1.00 N/mm, which was selected for following studies.

### 3.2. Effect of Number of Revolutions on Fibrillation of Lyocell Fiber

**Figure 4:**
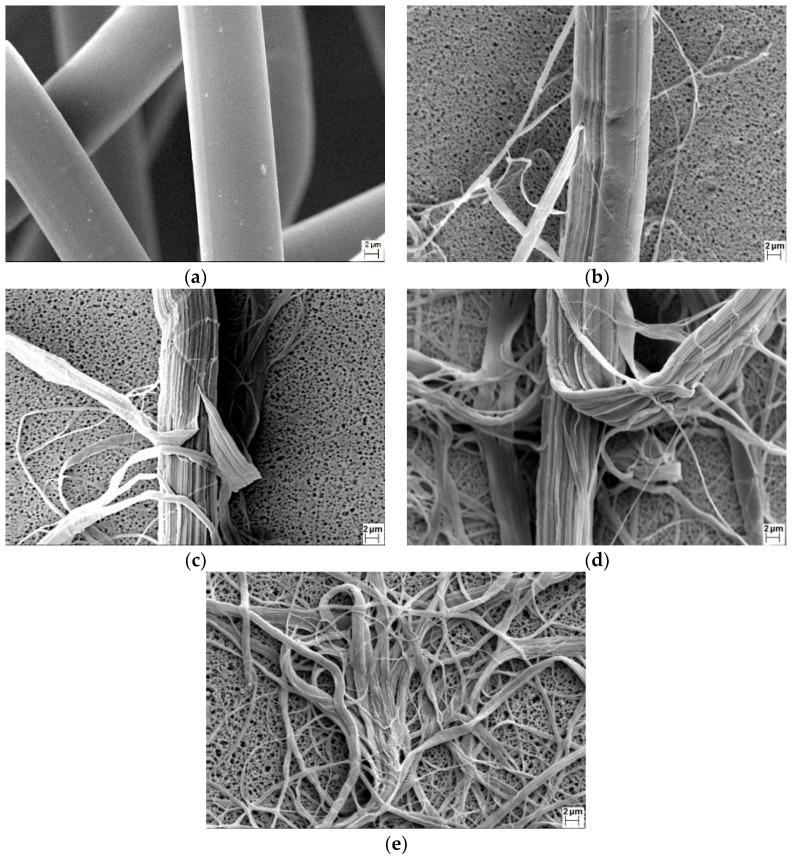
SEM images of fibrillated Lyocell fiber under a different number of revolutions: (**a**) original Lyocell fiber; (**b**) 5000 revolutions; (**c**) 10,000 revolutions; (**d**) 20,000 revolutions; (**e**) 40,000 revolutions.

The SEM images of the fibrillated fiber under a refining pressure of 1.00 N/mm can be seen in Figure 4, which demonstrated the fibrillation process. As shown in Figure 4a, the original Lyocell fiber was cylindrical, and the surface was very smooth. According to other studies [46], the Lyocell fiber had the island-in-the-sea structure and was made up of macrofibril (0.5–1 µm), while the macrofibril was made up of microfibril with diameter in the order of 100 nm. The Lyocell fiber with a skin-core structure had an amorphous skin of 35–80 nm [47]. In the initial stage of fibrillation, the skin of the Lyocell fiber was peeled off by the mechanical force of the PFI refiner, while the core of the Lyocell fiber was less affected by refining, as shown in Figure 4b. When the number of revolutions reached 10,000, most of the skin of the Lyocell fiber was peeled off, and individual or bundled macrofibrils were peeled off along the length of the Lyocell fiber, as can be seen in Figure 4c. When the number of revolutions reached 20,000, as shown in Figure 4d, more macrofibrils were peeled off, and the bundled macrofibrils were gradually split into individual macrofibrils. As the refining continued, most of the bundled macrofibrils were split into individual ones when the number of revolutions reached 40,000. The small amount of ultrafine fibers in Figure 4e indicated that small portions of macrofibrils were also split into microfibrils. The discussion of the fibrillation process showed that the cellulose nanofibers obtained under 1.00 N/mm and 40,000 revolutions were mainly the macrofibrils of the Lyocell fiber, and these nanofibers exhibited a range of fiber diameters.

The detailed properties of fibrillated fibers under different revolutions can be seen in Table 2. The beating degree increased from 13°SR to 79°SR when the number of revolutions increased. As the beating degree indicated the average fiber diameter, the tread of the beating degree agreed well with the changes of the fiber morphology in Figure 4. When the number of revolutions increased from 0 to 5000, the change of the beating degree was very small, as the fiber skin was peeled off but the core of the Lyocell fiber was less affected in this refining stage. When the number of revolutions was 10,000, the beating degree increased significantly as a result of the peeling of the macrofibrils from the Lyocell fiber. When 20,000 revolutions were reached, there was a sharp increase of the beating degree because of more individual macrofibrils. Under 40,000 revolutions, the beating degree continued to increase sharply, as most of the Lyocell fibers had been split into the fine macrofibrils.

It can also be found in Table 2 that the average fiber length decreased and the fine fibers content increased with the higher number of revolutions. It was noticeable that when the number of revolutions increased from 20,000 to 40,000, although the beating degree increased sharply, the changes of the average fiber length and the fine fiber content were small, which indicated that the refining energy was mostly used to spilt the bundled macrofibrils into finer fibrils in this refining stage. Because of the high longitudinal orientation of the crystallites of the Lyocell fiber [28,29,30], the average fiber length of the fibrillated nanofiber after 40,000 revolutions was more than 1 mm, which was much larger than the natural fiber under the same fibrillation condition. The specific surface area in Table 2 was used to calculate the average fiber diameter of the fibrillated fibers. As the fibrillated fibers from 0 to 10,000 revolutions owned a small specific surface area (smaller than 1.0 m^2^/g), it was very difficult to obtained accurate and reproducible results using the BET method. Only the specific surface area of the fibers under 20,000 and 40,000 are shown in Table 2. As can be seen, the average fiber diameter of the nanofibers under 40,000 were close to the diameter of the Lyocell microfibril (0.5–1 µm). It demonstrated that the cellulose nanofibers that were obtained under 40,000 revolutions were mainly macrofibrils of the Lyocell fiber, which agreed with the observation of the SEM images in Figure 4. As the Lyocell fiber was fully fibrillated with a good fiber length under 1.00 N/mm and 40,000 revolutions, the cellulose nanofibers under this condition were used to prepare the filter paper for the following tests.

### 3.3. Physical Properties of Filter Paper Containing Fibrillated Nanofiber

The physical properties of the filter paper containing different amounts of nanofibers are shown in Table 3. As can be seen, with the higher fraction of nanofibers, both the thickness and porosity slightly decreased, which showed that the cellulose nanofiber had little effect on the thickness and porosity of the filter paper. The mean pore size decreased dramatically with only a 5% nanofiber. Therefore, the nanofiber can reduce the pore size of the filter paper very efficiently. The mean pore size for the filter with a 20% nanofiber was 5.0 µm, which was larger than the PM_2.5_. It indicated that sieving was not a main filtration mechanism in this work, and that the particles were supposed to be captured by coarse fibers and nanofibers due to impaction, direct interception, and diffusion.

**Figure 5:**
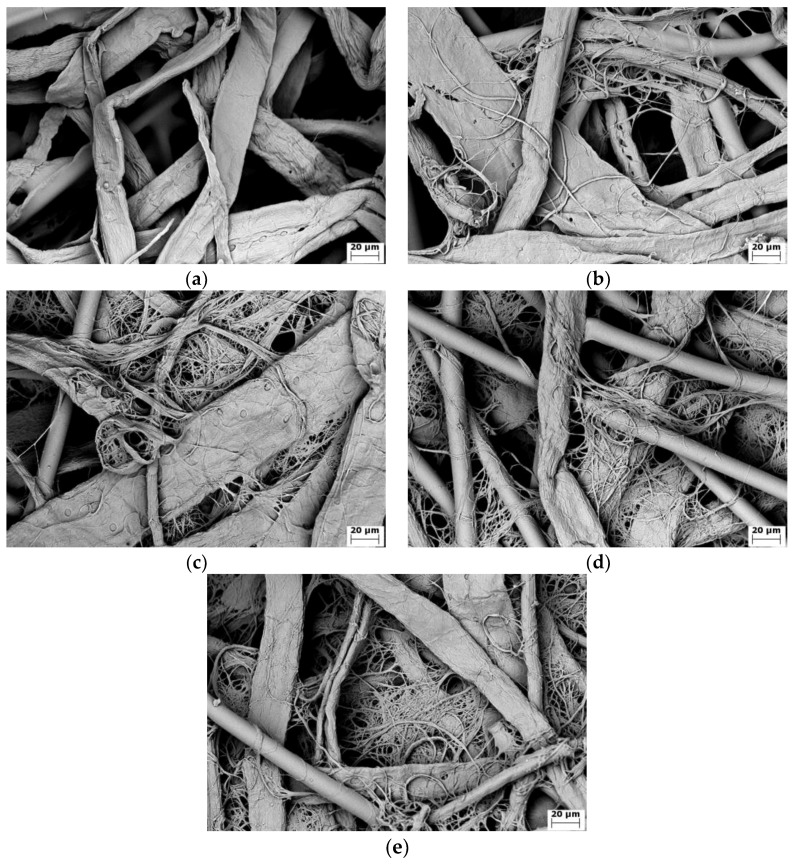
SEM images of filter paper containing different amounts of nanofibers: (**a**) sample #1; (**b**) sample #2; (**c**) sample #3; (**d**) sample #4; (**e**) sample #5.

The fiber microstructure of the filter paper containing different amounts of nanofibers are shown in Figure 5. For the filter paper without nanofibers, the fibrous structure was very open. With the presence of nanofibers in the filter paper, the fibrous structure greatly changed. As can be observed from Figure 5b to Figure 5e, there were two main ways of nanofiber arrangement in the filter paper, entangling with a coarse fiber or bridging the pores among the coarse fibers. When the nanofibers were entangled with the coarse fibers, the nanofibers would not contribute significantly to the filtration efficiency as expected. When the nanofiber bridged the pores, it would take advantage of the fine fiber size and lead to a good filtration performance. Therefore, from the perspective of filtration, it was preferred that the nanofibers were not entangled with other fibers. The impact of the nanofiber structure on the filtration performance is discussed in the next section.

### 3.4. Filtration Performance of Filter Paper Containing Fibrillated Nanofiber

**Figure 6:**
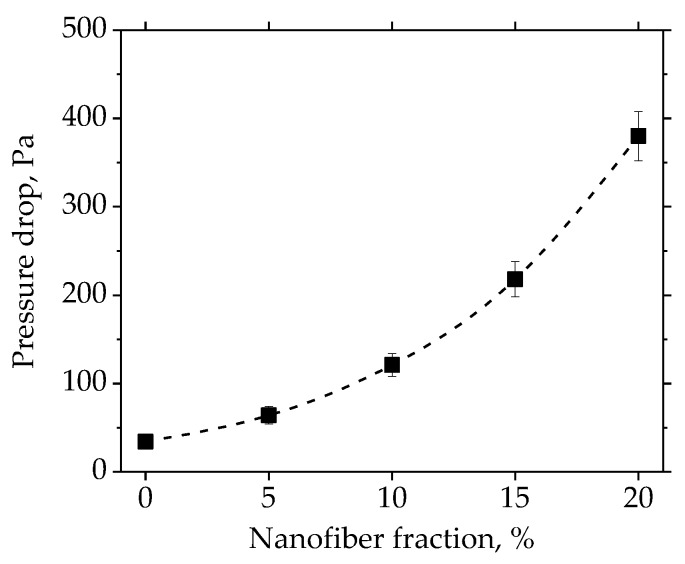
Pressure drop of filter paper with different fractions of nanofibers.

**Figure 7:**
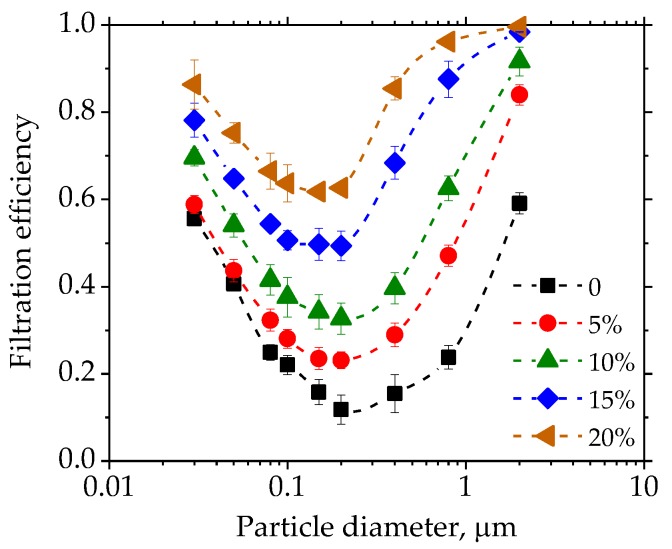
Fractional efficiency of filter paper with different fractions of nanofibers.

The pressure drop and fractional efficiency are critical parameters for the filter paper. The pressure drop of the filter paper with a different fraction of nanofibers is shown in Figure 6. The pressure drop increased with the higher fractions of nanofibers. When the nanofiber fraction reached 15%, the pressure drop increased sharply. The fractional efficiency of the different filter papers can be seen in Figure 7. The efficiency against the particles ranging from 30 nm to 2 µm increased gradually with the higher nanofiber fraction. The particle size at which the efficiency was lowest in the efficiency curve was the most penetration particle size (MPPS). The MPPS for all five of the cases was about 200 nm. The efficiency at the MPPS increased from 0.1 to 0.6, which indicated that the cellulose nanofiber made by the Lyocell fiber was effective in improving the filtration efficiency of the filter paper.

**Figure 8:**
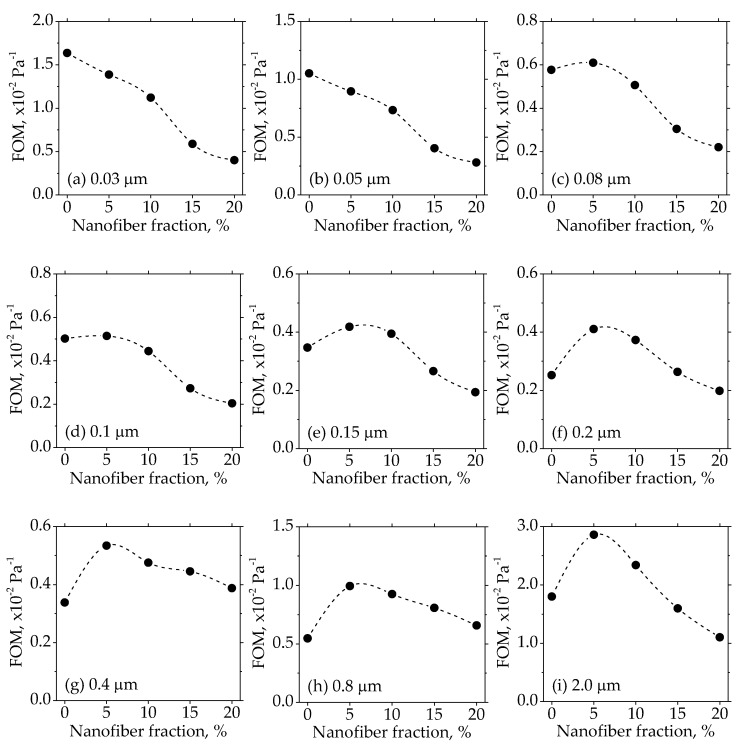
Figure of merit (FOM) of the filter paper against particles with different diameters.

To clarify the effects of the nanofiber fractions on the filtration performance, the figure of merit was used. The figure of merit was defined as the ratio of the efficiency per unit of thickness to the pressure drop per unit thickness [48], which meant that the only factor affecting the figure of merit was the difference of the fibers. The calculated results of the figure of merit of the filter paper with a different nanofiber fraction against the particles ranging from 0.03 µm to 2 µm, are shown in Figure 8. For the particles smaller than 0.05 µm, the nanofiber had an adverse effect on the figure of merit. This result agreed with the study of Jing et al. [11], whose theoretical calculations showed that the figure of merit decreased as the fiber size decreased for the very small particles (below 50 nm). It was because the dominant filtration mechanism for the particles smaller than 0.05 µm was the Brownian diffusion [5,49], and the coarse fiber had a better performance in the diffusion regime. For the particles larger than 0.05 µm, the figure of merit increased substantially when only 5% of the nanofiber was used, as the filtration mechanism of interception and impaction [5,49], on which the fiber size had a significant effect, became more important. However, when the nanofiber fraction reached 10% and higher, the figure of merit began to decrease gradually. As the particles larger than 0.5 µm contributed to a major part of the PM_2.5_ [43], the optimal fraction of the cellulose nanofiber against the particles in the PM_2.5_ regime was 5%. The results of the figure of merit were related to the inhomogeneous distribution of the nanofibers in the fibrous structure, based on the SEM images of Figure 5. As mentioned above, the nanofiber that bridges the pores among the coarse fibers was better than the entangling with coarse fibers. When the nanofiber fraction increased, it was more likely that the nanofibers formed fiber clusters and were entangled with the coarse fibers. Therefore, the proper way to use the cellulose fiber in the wet-laid paper was to keep the nanofiber fraction low.

### 3.5. Theoretical Analysis of Nanofiber Inhomogeneity

To characterize the inhomogeneity of the nanofibers in filter paper, Kirsch et al. [50] introduced the discrepancy between the theoretical and experimental pressure drop as the indicator of fiber inhomogeneity. For the theoretical calculation, the pressure drop across a filter paper can be calculated as the sum of the drags on all of the fibers. As the nanofibers were used, a gas slip effect should be considered. Pich [51] proposed Equation (5) to calculate pressure drop (Δ*p*, Pa), by using the Kuwabara field to account for the slip effect at the gas-fiber interface.

(5) $$\Delta p=\frac{16\mu lU_{0}\alpha \left(1+1.996Kn\right)}{d_{f}^{2}\left[Ku+1.996Kn\left(-0.5\alpha -0.25+0.25\alpha^{2}\right)\right]}$$

where *µ* is the air viscosity, Pa·s; *l* is the thickness of the filter paper, m; *U*_0_ is the air face velocity, m/s; *α* is the fiber packing density (*α*=1−*ε*); and *d_f_* is the average fiber diameter, m. *Kn* and *Ku* are Knudson number and Kuwabara number, and they can be calculated by Equations (6) and (7), respectively.

(6) $$Kn=\frac{2\lambda }{d_{f}}$$

(7) $$Ku=-0.5\ln\alpha -0.75+\alpha -0.25\alpha^{2}$$

where *λ* is the mean free path of air, m.

**Figure 9:**
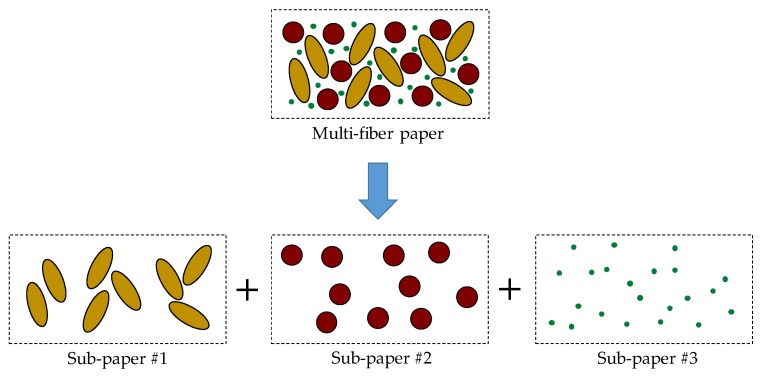
Schematic of multi-fiber paper and sub-papers.

Equation (5) can be used for the filter paper with a uniform fiber diameter. The average fiber diameter for the nanofiber/microfiber mixture, however, was difficult to determine. In order to calculate the theoretical pressure drop of the multi-fiber paper, this study proposed the following method. The multi-fiber paper can be considered as the combination of several sub-papers made by the same fiber, as shown in Figure 9.

The thickness of each sub-paper was the same as the multi-fiber filter paper. The fiber packing density of the sub-papers was determined by the volume fraction of each fiber in the multi-fiber filter paper, *f_V,i_*, which can be calculated using Equation (8), as follows:

(8) $$f_{V,i}=\frac{f_{m,i}/\rho_{i}}{\sum_{k=1}^{3}\left(f_{m,k}/\rho_{k}\right)}$$

where the subscript *i* (*i*=1,2,3) refers to the softwood fiber, PET fiber, and cellulose nanofiber, respectively; *f_m,i_* is the weight fraction of each fiber in the multi-fiber filter paper, as shown in Table 1; and *ρ_i_* is the density of each fiber, kg/m^3^.

The fiber packing density of each fiber, which equals the fiber packing density of the corresponding sub-paper, can be calculated by Equation (9), as follows.

(9) $$\alpha_{i}=f_{V,i}\left(1-\varepsilon \right)$$

The theoretical pressure drop of the sub-paper can be calculated by applying Equation (9) into Equation (5). The overall pressure drop of the multi-fiber filter paper is the sum of the pressure drops of all of the sub-papers, as shown in Equation (10).

(10) $$\Delta p=\Delta p_{1}+\Delta p_{2}+\Delta p_{3}$$

**Figure 10:**
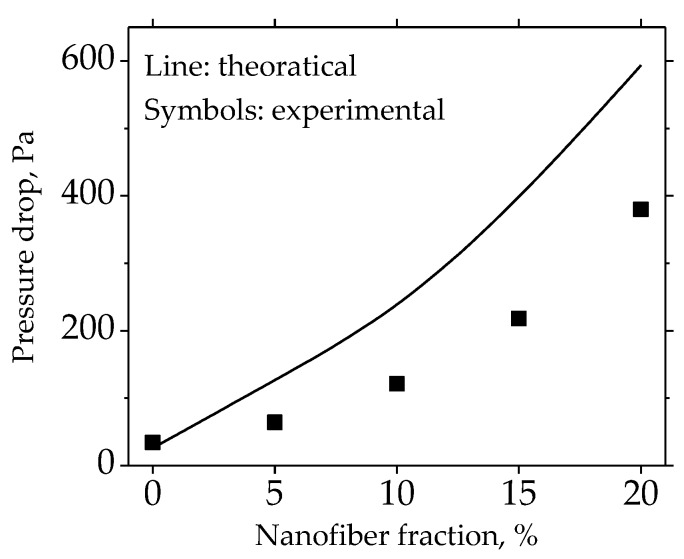
Experimental and theoretical pressure drop of filter paper.

The comparison of the theoretical and experimental results is shown in Figure 10. For the nanofiber fractions of 0 and 5%, the theoretical value was close to the experimental result. With the higher nanofiber fraction, the discrepancy between the theoretical and experimental results became larger. As the nanofibers were the major contributors of the pressure drop, they represented a higher degree of fiber inhomogeneity. As mentioned above in the fibrous structure analysis, the higher nanofiber fraction was more likely to result in fiber clusters and the entanglement with coarse fibers, which meant a lower nanofiber fiber inhomogeneity. The theoretical analysis agreed with the fibrous structure analysis, which confirmed that the optimal nanofiber fraction of 5% was due to the improved nanofiber homogeneity. As the filter papers in this study were prepared by standard procedures, some works will need to be done in the future in order to study the possibility of increasing the nanofiber homogeneity in the wet-laid preparation process, so as to maximize the filtration advantages of the nanofibers when the nanofiber fraction is higher.

## 4. Conclusions

In this work, cellulose nanofibers were prepared by Lyocell fiber nanofibrillation, and the influence of applying the cellulose nanofibers on filter paper was studied. It was found that the refining pressure of 1.00 N/mm was more satisfactory to fibrillate the Lyocell fiber, while the fibers were largely cut under the standard refining pressure of 3.33 N/mm. In the nanofibrillation process, the skin of the Lyocell fiber was peeled off first. Then, the individual or bundled macrofibrils were peeled off along the length of the Lyocell fiber. With further fibrillation, more macrofibrils were peeled off, and the bundled macrofibrils were gradually split into individual macrofibrils. The analysis of the fibrillation process showed that the cellulose nanofibers obtained under 1.00 N/mm and 40,000 revolutions were mainly macrofibrils of the Lyocell fiber with an average fiber diameter of 0.8 µm, which was suitable for filter paper. For the filter paper containing different fractions of cellulose nanofibers, both the pressure drop and fractional efficiency increased with a higher fraction of nanofibers. The results of the figure of merit demonstrated that for the particles smaller than 0.05 µm, the nanofiber had an adverse effect on the figure of merit, while for the particles larger than 0.05 µm, the figure of merit increased substantially with 5% nanofibers, but decreased when the nanofiber fraction reached 10% and higher. It was concluded that the optimal fraction of the cellulose nanofiber against the PM_2.5_ was 5%. The results of the figure of merit were related to the inhomogeneous distribution of the nanofiber in the fibrous structure. Based on the SEM observation, when the nanofiber fraction increased, it was more likely that the nanofibers formed fiber clusters and entangled with the coarse fibers. The theoretical analysis of the nanofiber inhomogeneity indicated that the higher nanofiber fraction led to a higher degree of fiber inhomogeneity, which confirmed that the optimal nanofiber fraction of 5% was due to an improved nanofiber homogeneity.

## Figures and Tables

**Table 1:** Weight fraction of fibers in filter paper.

Sample No.	Softwood Fiber	PET Fiber	Lyocell Nanofiber
#1	75%	25%	0
#2	70%	25%	5%
#3	65%	25%	10%
#4	60%	25%	15%
#5	55%	25%	20%

**Table 2:** Properties of fibrillated fibers under different revolutions.

Number of Revolutions	Beating Degree, °SR	Average Fiber Length, mm	Fine Fibers Content, %	Specific Surface Area, m^2^/g	Average Fiber Diameter *d_f_*, µm
0	13	4.00 ± 0.01	0	-	-
5000	18	3.16 ± 0.06	15.0 ± 0.2	-	-
10,000	31	2.66 ± 0.08	29.8 ± 1.1	-	-
20,000	58	1.47 ± 0.11	38.3 ± 1.2	2.42 ± 0.04	1.1 ± 0.09
40,000	79	1.31 ± 0.09	43.7 ± 1.5	3.33 ± 0.02	0.799 ± 0.06

**Table 3:** Physical properties of filter paper containing nanofiber.

Filter Paper No.	Weight Fraction of Nanofibers	Basic Weight, g/m^2^	Thickness, mm	Porosity	Mean Pore Size, µm
#1	0	104.3 ± 1.0	0.452 ± 0.012	0.843 ± 0.020	22.6 ± 0.5
#2	5%	104 ± 1.5	0.408 ± 0.010	0.827 ± 0.013	13.5 ± 0.3
#3	10%	103.6 ± 1.3	0.415 ± 0.009	0.830 ± 0.023	10.2 ± 0.3
#4	15%	103.6 ± 1.6	0.400 ± 0.010	0.824 ± 0.031	6.1 ± 0.2
#5	20%	104 ± 0.9	0.390 ± 0.011	0.819 ± 0.021	5.0 ± 0.2

## Supplementary Materials

The following are available online at http://www.mdpi.com/1996-1944/11/8/1313/s1, Table S1: Technical data sheet of Lyocell fiber.
