# Supplementary material for: Preparation of Fibrillated Cellulose Nanofiber from Lyocell Fiber and Its Application in Air Filtration
Source: Materials (Basel). 2018 Jul 29;11(8):1313. doi: 10.3390/ma11081313 (PMC6117698; doi:10.3390/ma11081313)
Supplement: Supplementary file 1 [file materials-11-01313-s001.pdf]

Table S1. Technical data sheet of Lyocell fiber

Properties	Value
Standard Filament Size	1.7 dtex
Standard Cut Lengths	4 mm
Cross-section	Round
Luster	Bright, uncrimped
Specific Gravity	1.53 g/cm^3
Whiteness	63 CIE
Finish level	0.22%
Dry Tenacity	4.5 - 5.0 g/den
Elongation at break, dry	24% – 26%
Wet Tenacity	3.9 - 4.3 g/den
Elongation at break, wet	16% - 18%
Degree of polymerization	550 - 600
Initial Moisture Content	10%
Dispersibility in water	Very good
